# MicroRNA519d and microRNA4758 can identify gangliogliomas from dysembryoplastic neuroepithelial tumours and astrocytomas

**DOI:** 10.18632/oncotarget.25563

**Published:** 2018-06-15

**Authors:** Anika Bongaarts, Avanita S. Prabowo, Andrea Arena, Jasper J. Anink, Roy J. Reinten, Floor E. Jansen, Wim G.M. Spliet, Maria Thom, Roland Coras, Ingmar Blümcke, Katarzyna Kotulska, Sergiusz Jozwiak, Wieslawa Grajkowska, Figen Söylemezoğlu, José Pimentel, Antoinette Y.N. Schouten-van Meeteren, James D. Mills, Anand M. Iyer, Erwin A. van Vliet, Angelika Mühlebner, Eleonora Aronica

**Affiliations:** ^1^ Department of (Neuro)Pathology, Academic Medical Center, University of Amsterdam, Amsterdam, The Netherlands; ^2^ Department of Biochemical Sciences, Sapienza University of Rome, Rome, Italy; ^3^ Department of Pediatric Neurology, University Medical Center Utrecht, Utrecht, The Netherlands; ^4^ Department of Pathology, University Medical Center Utrecht, Utrecht, The Netherlands; ^5^ Neuropathology Department, University College London, Institute of Neurology, London, UK; ^6^ Department of Neuropathology, University Hospital Erlangen, Erlangen, Germany; ^7^ Department of Neurology and Epileptology, Children's Memorial Health Institute, Warsaw, Poland; ^8^ Department of Child Neurology, Medical University of Warsaw, Warsaw, Poland; ^9^ Department of Pathology, Children's Memorial Health Institute, Warsaw, Poland; ^10^ Department of Pathology, Faculty of Medicine, Hacettepe University, Ankara, Turkey; ^11^ Department of Neurology, Hospital de Santa Maria, Lisbon, Portugal; ^12^ Department of Pediatric Oncology, Emma Children's Hospital, Academic Medical Center, University of Amsterdam, Amsterdam, The Netherlands; ^13^ Department of Pediatrics, Medical University of Vienna, Vienna, Austria; ^14^ Stichting Epilepsie Instellingen Nederland, Heemstede, The Netherlands

**Keywords:** low-grade epilepsy-associated brain tumours, epilepsy, glioneuronal tumour, ganglioglioma, dysembryoplastic neuroepithelial tumour

## Abstract

Glioneuronal tumours, including gangliogliomas and dysembryoplastic neuroepithelial tumours, represent the most common low-grade epilepsy-associated brain tumours and are a well-recognized cause of intractable focal epilepsy in children and young adults. Classification is predominantly based on histological features, which is difficult due to the broad histological spectrum of these tumours. The aim of the present study was to find molecular markers that can be used to identify entities within the histopathology spectrum of glioneuronal tumours. The focus of this study was on microRNAs (miRNAs). miRNAs are important post-transcriptional regulators of gene expression and are involved in the pathogenesis of different neurological diseases and oncogenesis. Using a miRNA array, miR-519d and miR-4758 were found to be upregulated in gangliogliomas (n=26) compared to control cortex (n=17), peritumoural tissue (n=7), dysembryoplastic neuroepithelial tumours (n=9) and astrocytomas (grade I-IV; subependymal giant cell astrocytomas, n=10; pilocytic astrocytoma, n=15; diffuse astrocytoma grade II, n=10; grade III, n=14 and glioblastoma n=15). Furthermore, the PI3K/AKT3/P21 pathway, which is predicated to be targeted by miR-519d and miR-4758, was deregulated in gangliogliomas. Functionally, overexpression of miR-519d in an astrocytic cell line resulted in a downregulation of *CDKN1A* (P21) and an increase in cell proliferation, whereas co-transfection with miR-4758 counteracted this effect. These results suggest that miR-519d and miR-4758 might work in concert as regulators of the cell cycle in low grade gliomas. Furthermore, these miRNAs could be used to distinguish gangliogliomas from dysembryoplastic neuroepithelial tumours and other low and high grade gliomas and may lead to more targeted therapy.

## INTRODUCTION

Low-grade epilepsy-associated brain tumours (LEATs), including glioneuronal tumours (GNTs) such as gangliogliomas (GGs) and dysembryoplastic neuroepithelial tumours (DNTs), represent the most frequent tumour entity in young patients who undergo surgery for chronic intractable focal epilepsy [1–4].

Patients with LEATs often have a history of 2 or more years of drug-resistant epilepsy [5]. GGs and DNTs are low grade, stationary or very slow growing, cortical based tumours with a very low risk of tumour recurrence and malignant progression [3, 5]. These tumours often present with early seizure onset at a mean age of 16.5 years [1, 2]. In the majority of cases surgical resection shows favourable prognosis, both in terms of tumour management and seizure outcome. However, in a small proportion of cases, seizures may persist [2, 3, 6].

The histopathological features of GNTs include a mixture of neuronal (dysplastic neurons) and glial elements in GGs, while a specific glioneuronal element is evident in DNTs [3, 7]. Immature neural elements combined with expression of stem cell markers and the coexistence with cortical dysplasia suggest a developmental pathogenesis for GGs and DNTs. This is supported by studies indicating that these tumours are associated with molecular alterations of key developmental signalling pathways, including the enhanced activation of the mitogen-activated protein kinase/extracellular signal-regulated kinase (MAPK/ERK) and the phosphoinositide 3-kinase/Protein Kinase B/mechanistic target of rapamycin (PI3K/AKT/mTOR) pathway [1, 8–11]. The *BRAF* c.1799T>A (p.V600E; *BRAF V600E*) mutation was found to be a common genetic driver in GGs (18-56%). However, the frequency of this mutation varies considerably between studies [8, 12–15]. In other LEATs, the *BRAF* mutation has been observed in 30-51% of DNTs [13, 15, 16] and in 3/10 of the recently described polymorphous low-grade neuroepithelial tumours of the young (PLNTY) [17], but not in multinodular and vacuolating neuronal tumours (MVNT) [18] and angiocentric gliomas [19]. In other low grade gliomas such as pleomorphic xanthoastrocytoma (PXA) and pilocytic astrocytomas (PA) the *BRAF V600E* mutation has also been observed [8, 20, 21]. Therefore, the presence of the *BRAF V600E* mutation cannot be regarded as specific for any tumour entity. In contrast to the *BRAF V600E* mutation, tyrosine kinase activating *FGFR1* gene mutations were found more frequently in DNTs (58-82%) [9, 19], suggesting this mutation could be a good marker for DNTs. However, besides the *FGFR1* mutations, *BRAF* mutations and copy number abnormalities also occur in DNTs, making it difficult to distinguish between GGs and DNTs based only on the presence of *BRAF* and *FGFR1* mutations [13, 15, 16, 22].

The 2016 revised WHO classification for LEATs is based on histological criteria, but does not involve an integration of molecular and pathological analysis techniques [1]. Although, several histological classification systems have been proposed [1–3], the broad spectrum of LEATs renders classification using these systems very difficult. Recently, a molecular classification was suggested for glioneuronal tumours, highlighting the importance of integrating molecular diagnostics in classifying these tumours [23, 24].

The aim of the present study was to find molecular markers that can be used to identify specific LEATs. The focus was on microRNAs (miRNAs), since they are important post-transcriptional regulators of gene expression, involved in the pathogenesis of different neurological diseases and oncogenesis [25–28]. In particular we investigated the expression and function of miR-519d and miR-4758, two miRNAs involved in the regulation of the PI3K/AKT3/P21 pathway, that could be used to distinguish GGs from DNTs and other low and high grade gliomas.

## RESULTS

### Mutation analysis of GG and DNT

The *BRAF V600E* mutation was found in 16 of the 26 (61.5%) GG samples. An initial screen using sanger sequencing identified 11 GG samples that were positive for the *BRAF V600E* mutation. Samples that were deemed *BRAF V600E* negative based on sanger sequencing and of which a sufficient amount of DNA was still available were screened further using a next-generation sequencing panel. Based on this panel an additional 5 GG samples were found to be positive for *BRAF V600E*. One *BRAF V600E* positive GG also had an additional BRAF mutation (*BRAF T559R*) on the same allele. A total of 8 DNTs were also screened on the panel of which 1 was found positive for the *BRAF V600E*, 1 was found positive for *FGFR1* mutations (*FGFR1 G539R* and *FGFR1 K656E*) and 1 was found positive for *CIC G935R* mutation of which the pathological implication is unknown.

### miR-519d and miR-4758 expression in GG

**Figure 1:**
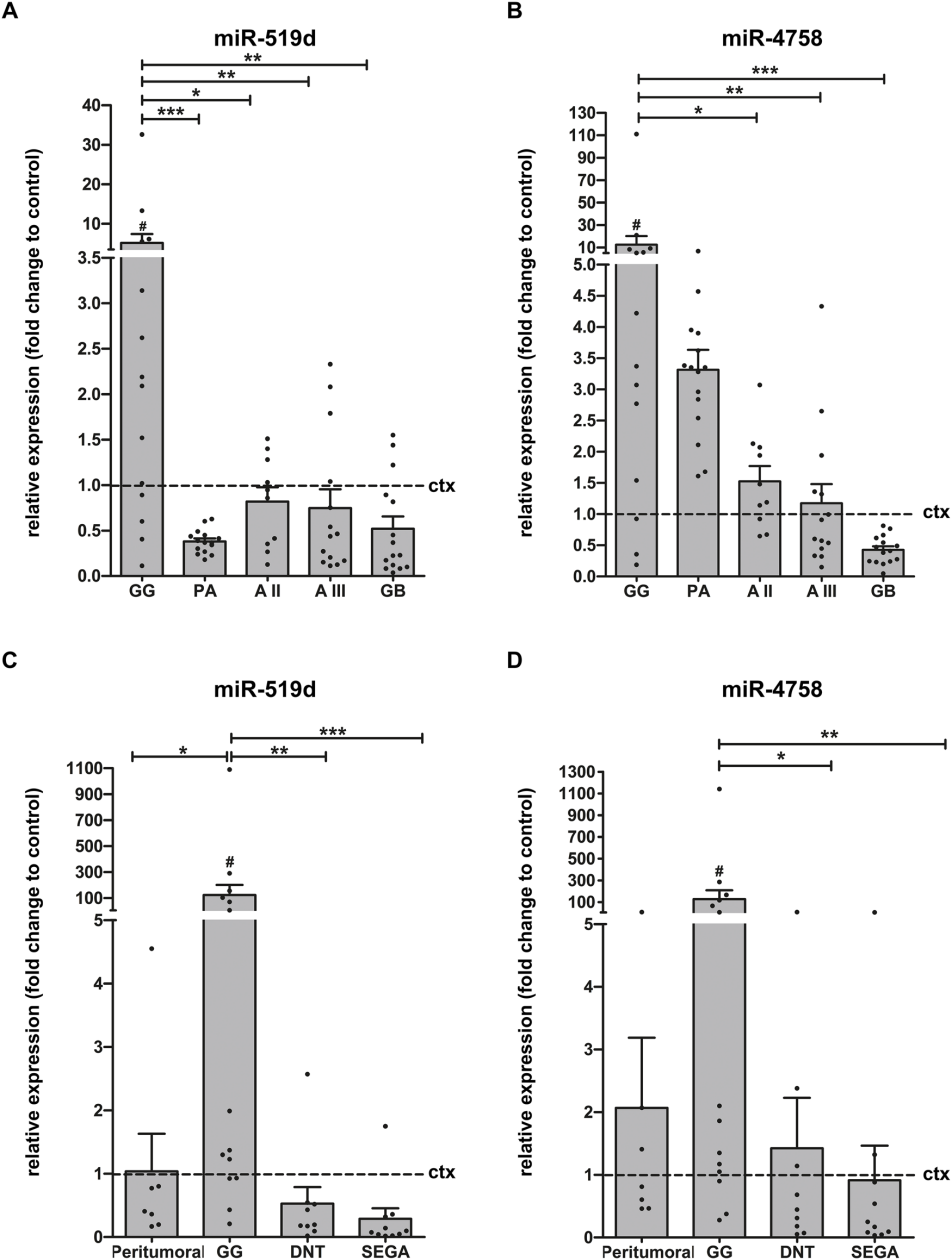
Relative expression of miR-519d and miR-4758 in GNTs and astrocytomas Quantitative real-time PCR. Panels **(A** and **B)**: Relative expression of miR-519d (A) and miR-4758 (B) in GG (n=14), PA (n=15), AII (n=10), AIII (n=14) and GB (n=15). Data are expressed relative to the expression in control cortex (n=7; frozen material). Panels **(C** and **D)**: Expression of miR-519d (C) and miR-4758 (D) in peritumoural GG (n=7), GG (n=14), DNT (n=9) and SEGA (n=10); FFPE. Data are expressed relative to the expression in control cortex (n=9). miRNA expression was normalized to that of the U6B small nuclear RNA gene (*Rnu6B*) in all cohorts. Dots represent individual samples. The error bars represent SEM. ^*^p < 0.05; ^**^p < 0.01, ^***^p < 0.001 between experimental samples, Kruskal-Wallis test followed by Mann-Whitney U test; #p < 0.05 between GG and control cortex, Mann-Whitney U test. ctx: control cortex; GG: ganglioglioma; PA: pilocytic astrocytoma; AII: astrocytoma grade II; AIII: astrocytoma grade III; GB: glioblastoma; DNT: dysembryoplastic neuroepithelial tumour; SEGA: subependymal giant cell astrocytoma.

First, we performed a global expression analysis of 1891 miRNAs using an Exiqon miRCURY LNA™ microRNA Array on 11 frozen brain samples (control cortex, n=5; GGs, n=6). Compared to control cortex, a total of 5 miRNAs: miR-519d, miR-4758, miR-664b, miR-4714 and miR-5681b were differentially expressed in GGs. We further validated these 5 miRNAs using Taqman miRNA assays in a larger cohort (frozen samples, control cortex, n=7; GG, n=14). Only miR-519d and miR-4758 were confirmed to be differentially expressed in GGs compared to control cortex, showing 5-fold and 13-fold upregulation (p=0.0335 and p=0.0153, respectively; Figure 1A-1B). Receiver operating characteristic (ROC) analysis for miR-519d yielded an area under the curve (AUC) of 0.796, p=0.031 and for miR-4758 AUC=0.837, p=0.0139. Furthermore, a positive correlation was observed between the two miRNAs (r=0.731, p=0.0002). We also evaluated the expression of both miRNAs in GG compared to other gliomas, including PA (n=15), diffuse astrocytoma grade II (AII; n=10), diffuse astrocytoma grade III (AIII; n=14) and glioblastoma (GB; n=15; Figure 1A-1B). miR-519d was upregulated in GG compared to all other gliomas, whereas miR-4758 was upregulated compared to AII, AIII, GB but not PA. Furthermore, no difference was found in expression of both miRNAs in *BRAF V600E* mutated GGs (n=8) compared to wildtype GGs (n=6; p=0.950 and p=0.414; data not shown).

### miR-519d and miR-4758 expression by quantitative real-time PCR in GG, DNT and subependymal giant cell astrocytoma

Recent studies indicate a robust stability of miRNAs, supporting the accuracy of miRNA measurements with quantitative real-time PCR, also in formalin-fixed paraffin-embedded (FFPE) tissues [29–31]. Therefore, miR-519d and miR-4758 expression was also evaluated in a large cohort of FFPE samples, including control cortex (n=9), GG (n=14), peritumoural cortex (n=7), DNT (n=9) and subependymal giant cell astrocytoma (SEGA; n=10) samples. Normal appearing peritumoural tissue represents appropriate disease control tissue for GG, since it is exposed to the same seizure activity, drugs and fixation time, while also being of identical age and gender. Evaluation of miR-519d and miR-4758 expression in this cohort confirmed the upregulation of miR-519d in GG compared to control cortex (p=0.041), DNT, SEGA and peritumoural cortex, whereas miR4768 was upregulated in GG compared to control cortex (p=0.035), DNT and SEGA but not when compared to peritumoural cortex (Figure 1C-1D).

### miR-519d and miR-4758 cellular distribution by *in situ* hybridization in GG

**Figure 2:**
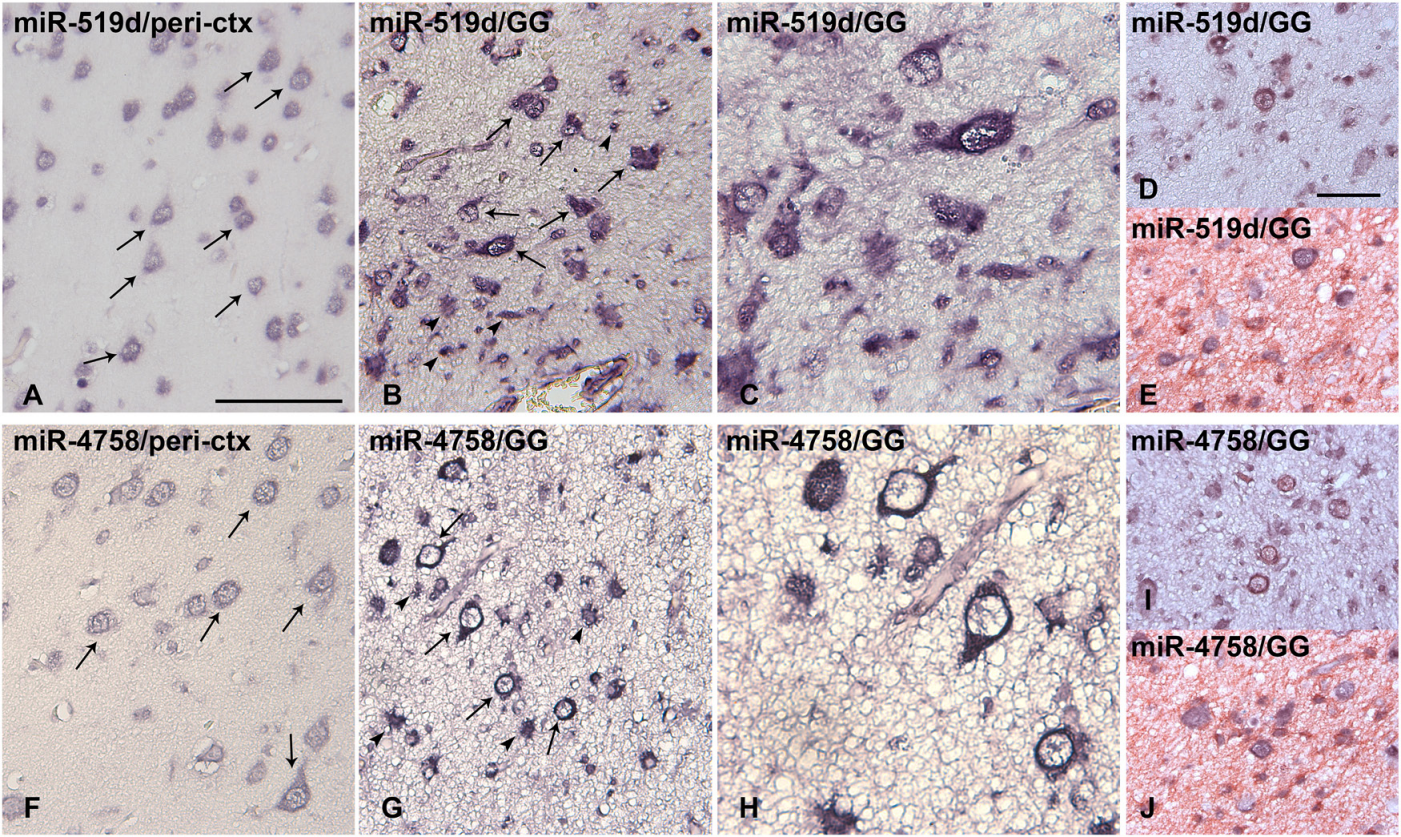
*In situ* hybridization of miR-519d and miR-4758 expression in peritumoural cortex and ganglioglioma Panels **(A** and **F)**: Normal appearing peritumoural cortex (ctx); miR-519d (A) and miR-4758 (F) were weakly expressed in neurons (arrows) and could not be detected in glial cells. Panels **(B-C)** and **(G-H)**: GG; miR-519d (B-C) and miR-4758 (G-H) were expressed in both the neuronal (arrows) and the glial (arrowheads) tumour components; Panels D and I show expression of miR-519d (D) and miR-4758 (I) in neurons (NeuN-positive, red) panels E and J show expression of miR-519d (E) and miR-4758 (J) in astrocytes (GFAP-positive, red) GG: ganglioglioma. Scale bar in A: 100 μm in A-B and F-G, 50 μm in C and H; scale bar in D: 50 μm in D-E and I-J.

The cellular distribution of miR-519d and miR-4758 in peritumoural tissue (n=6) and GG (n=7) was investigated using *in situ* hybridization (ISH). In peritumoural tissue a weak expression of miR-519d and miR-4758 was observed in neuronal cells, but could not be detected in glial cells (Figure 2A and 2F). Expression of both miR-519d and miR-4758 was found specifically in cells with atypical astroglia morphology and in dysplastic neurons (Figure 2B-2C and 2G-2H). Double labelling confirmed miR-519d and miR-4758 expression in NeuN-positive neurons and GFAP-positive astrocytes in GG (Figure 2D-2E and 2I-2J).

### miRNA target expression in GG

**Figure 3:**
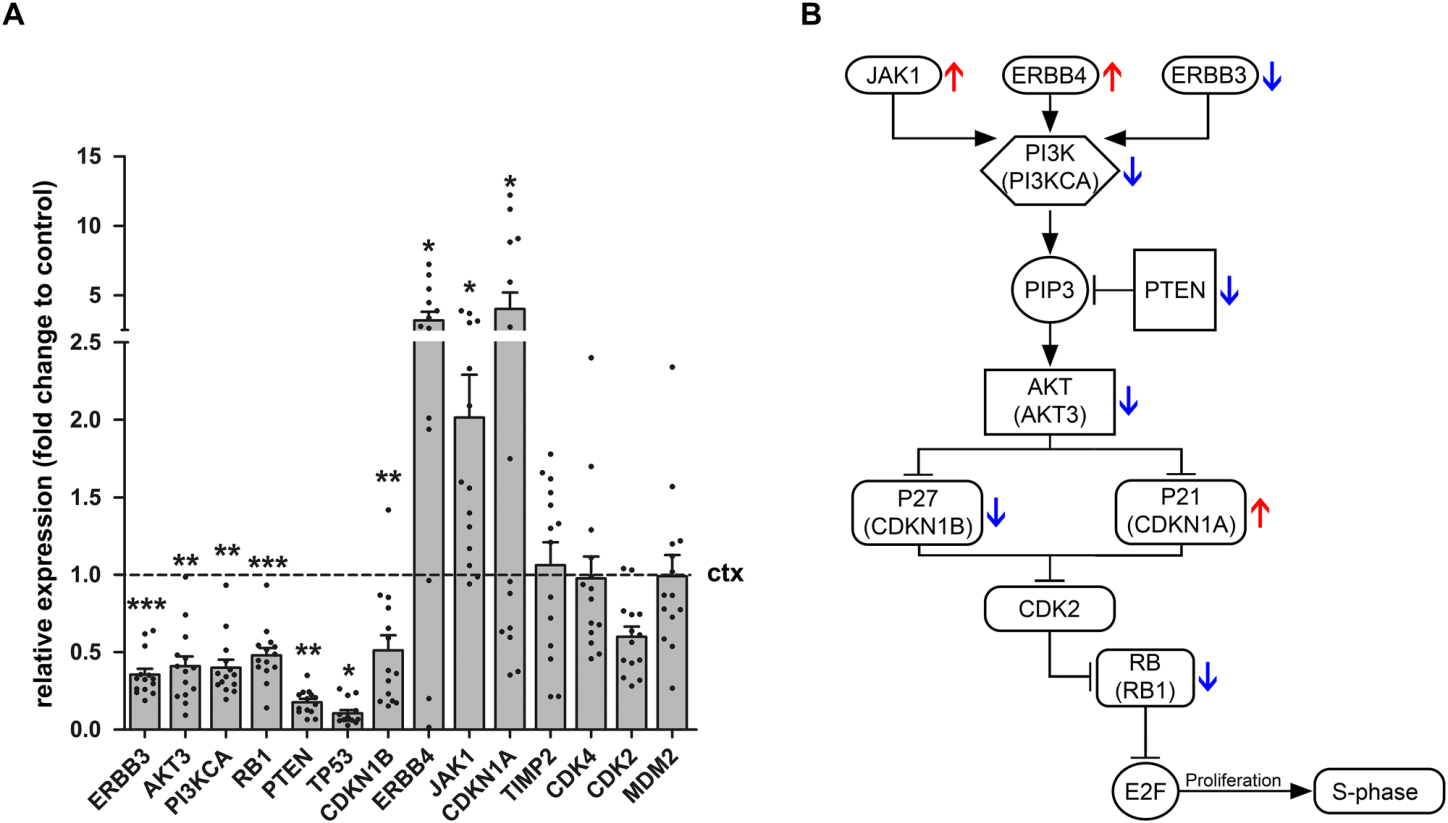
Relative expression of miR-519d and miR-4758 target genes in ganglioglioma Quantitative real-time PCR of *ERBB3*, *AKT3*, *PI3KCA*, *RB1*, *PTEN*, *TP53*, *CDKN1B*, *ERBB4*, *JAK1*, *CDKN1A*, *TIMP2*, *CDK4*, *CDK2* and *MDM2* in GG (n=14, except for *ERBB4* where n=13) compared to control cortex (n=7). Panel **(A)** shows increased mRNA expression of *ERBB4*, *JAK1* and *CDKN1A* and decreased mRNA expression of *ERBB3*, *AKT3*, *PI3KCA*, *RB1*, *PTEN*, *TP53* and *CDKN1B* in GG compared to control cortex. The expression of *TIMP2*, *CDK4*, *CDK2* and *MDM2* did not change. Data are expressed relative to the expression observed in control cortex. mRNA expression was normalized to that of *EF1*α. Dots represent individual samples. The error bars represent SEM; statistical significance: ^*^p < 0.05; ^**^p < 0.01, ^***^p < 0.001, Kruskal–Wallis test followed by Mann-Whitney U test. ctx: control cortex. GG: ganglioglioma. Panel **(B)** shows a schematic overview of the PI3K pathway. The red arrows indicate the upregulation of genes and the blue arrows the downregulation of genes as indicated in panel A.

In order to identify potential targets of miR-519d and miR-4758 5 different databases were utilised. Targets were considered relevant if found in at least 3 of the 5 databases. Based on these target prediction tools, *AKT3*, *CDKN1A* (P21), *JAK1*, *PTEN*, *ERBB3*, *ERBB4*, *RB1* and *TIMP2* were identified *a*s potential targets of miR-519d. Both clinical and experimental studies have shown the potential contribution of miR-519d dysregulation in hepatocellular carcinoma, breast cancer and cervical cancer [32–34]. *AKT3*, *CDKN1A* and *PTEN* have all been shown to be downregulated by miR-519d, potentially explaining the oncogenic features of this miRNA [35, 36]. Based on target prediction tools the relatively unknown miRNA, miR-4758 was predicted to target *CDK2* and *CDKN1B* (P27), raising the possibility that miR-4758 also has oncogenic properties. Evaluation of the mRNA expression of miR-519d and miR-4758 targets (Figure 3) showed a downregulation of *ERBB3* (p=0.0005), *AKT3* (p=0.0015), *PI3KCA* (p=0.0015), *RB1* (p=0.0009), *PTEN* (p=0.0041), *TP53* (p=0.023) and *CDKN1B* (p=0.0065) in GG (n=14, except for ERBB4 where n=13) compared to control cortex (n=7; Mann-Withney U test). Furthermore, targets *ERBB4* (p=0.032) *CDKN1A* (p=0.048) and *JAK1* (p=0.0124) were found to be upregulated in GG compared to control cortex. The expression of *TIMP2* (p=0.68), *CDK4* (p=0.39), *CDK2* (0.91) and *MDM2* (p=0.63) did not change.

### miRNA target expression in cell culture

**Figure 4:**
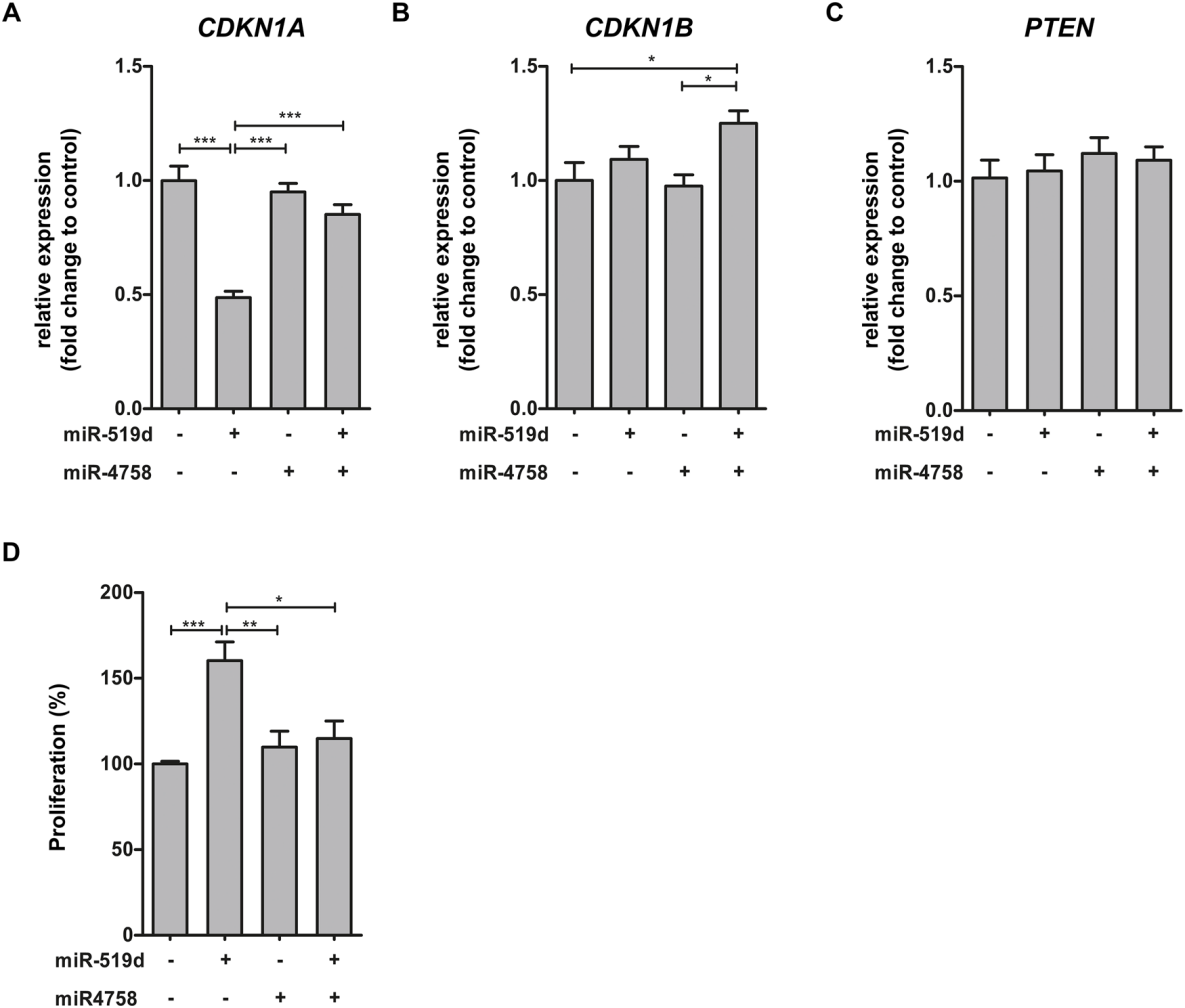
miR-519d and miR-4758 target mRNA expression and the regulation of cell cycle in cell culture Panels **(A-C)**: Relative expression of *CDKN1A* (A), *CDKN1B* (B) and *PTEN* (C) after transfection of LGG2 cell line for 24 hours with lipofectamine (n=5), miR-519d mimic (n=4), miR-4758 mimic (n=5) or miR-519d mimic co-transfected with miR-4758 mimic (n=5). Panel **(D)**: The S phase of cell cycle progression after transfection with lipofectamine (n=7), miR-519d (n=7), miR-4758 (n=8) and miR-519d (n=7) co-transfected with miR-4758 in LGG2. miR-519d transfected LGG2 cells displayed an increased S phase compared to the negative control. miR-4758 transfected cultures displayed no alteration in S phase. Co-transfection of miR-519d and miR-4758 was able to mimic the response of the negative control. The error bars represent SEM; statistical significance: ^*^p < 0.05; ^**^p < 0.01, ^***^p < 0.001, Kruskal-Wallis test followed by Mann-Whitney U test.

Given the lack of GG cell lines, we used the human pediatric low grade astrocytoma cell line Res259 (LGG2) obtained from a pediatric AII [37] to investigate the effects of miR-519d and miR-4758 upregulation. After transfection with miRNA mimic, overexpression of the specific miRNA was observed under basal condition (data not shown). Quantitative real-time PCR analysis was performed to evaluate the effects of miR-519d and miR-4758 transfection on mRNA expression of a subset of targets related to the PI3K/AKT pathway, involved in the regulation of cell cycle progression [38]. *CDKN1A* was downregulated after miR-519d transfection, whereas transfection with miR-4758 did not affect *CDKN1A* expression compared to control (Figure 4A). Co-transfection of miR-519d with miR-4758 rescued the effect of miR-519d transfection. The expression of miR-4758 target *CDKN1B* was not affected by transfection with miR-4758 or miR-519d alone (Figure 4B). However, after co-transfection with miR-4758 and miR-519d *CDKN1B* was upregulated compared to control. Furthermore, *PTEN* mRNA expression did not change after transfection with either one of the miRNAs (Figure 4C).

### Effects of miR-519d and miR-4758 modulation on the cell cycle

We further evaluated the role of miR-519d and miR-4758 on cell cycle progression using flow cytometry analysis in Res259/LGG2. Overexpression of miR-519d showed a decreased G_1_ phase (13%, *p<0.01*) whereas, transfection with miR-4758 resulted in an increased G_2_/M phase. Overexpression of miR-519d, but not miR-4758, increased the S phase; miR-4758 mimic co-transfected with miR-519d mimic was able to counteract the effect of miR-519d alone (Figure 4D).

## DISCUSSION

In this study, we showed that miR-519d and miR-4758 are specifically upregulated in GGs compared to control tissue, DNTs and other gliomas. In addition, we showed that the PI3K/AKT3/P21 pathway was deregulated in GGs. Importantly, we observed a downregulation of *CDKN1A* (P21) and an increase in cell proliferation after miR-519d overexpression in an astrocytic cell line, whereas co-transfection with miR-4758 counteracted this effect, suggesting an oncogenic function of miR-519d.

GNTs represent a major cause of medically intractable epilepsy in young patients, however their underlying biology remains to be fully investigated. The 2016 revised WHO classification includes histopathological criteria for LEATs [1, 7]. However, the different GNTs are difficult to distinguish by their histological features even by experienced pathologists [1, 3]. Improving the classification and the ability to distinguish LEATs from IDH1/2-wildtype low-grade gliomas is becoming more important to avoid unnecessary treatment with chemo-radiation therapy [1, 39]. Due to the current limitations in the classification of LEATs there is a need for developing accurate molecular techniques that can be used for diagnosis. In this study we evaluated the role of miRNAs in distinguishing GGs from DNTs and other gliomas. We found two miRNAs, miR-519d and miR-4758, upregulated in GGs compared to control tissue, DNTs and other gliomas. Therefore, these two miRNAs could be considered as additional markers in the classification of LEATs, especially in distinguishing GGs from DNTs and other low and high grade gliomas.

Recently, it was suggested that glioneuronal tumours can be separated into two groups: one group enriched for *BRAF* mutations with an astrocytic expression phenotype and one group enriched for *FGFR1* mutations with an oligodendrocyte precursor expression phenotype [23]. It would be interesting to see how miR-519d and miR-4758 are expressed in the two groups as suggested by Stone et al., 2018. Here, we evaluated the expression of both miRNAs in *BRAF* mutated GGs and GGs in which no mutation could be identified and we were unable to find any differences.

Using online prediction databases we identified the PI3K/AKT3/P21 pathway as a predicted target of both miR-519d and miR-4758. Next, we showed that the genes related to the PI3K/AKT3/P21 pathway were differentially expressed in GGs compared to control tissue, indicating that this pathway is deregulated in GGs. Overexpression experiments with miR-519d and miR-4758 indicate that overexpression of miR-519d increased proliferation, which could be explained by the downregulation of *CDKN1A*, a key player of the PI3K/AKT3/P21 pathway. This is in agreement with previous studies showing that downregulation of *CDKN1A*, a direct target of miR-519d, results in increased proliferation [35, 36, 40]. Overexpression of miR-4758 rescued the expression of *CDKN1A* and the associated increase in proliferation due to miR-519d, suggesting a tumour suppressive role for miR-4758. However, transfection of miR-4758 did not downregulate the predicted target gene *CDKN1B*, highlighting the limitations and the challenges posed by the interpretation of miRNA overexpression experiments to achieve insights into the function of specific miRNAs in the regulation of the cell cycle. Therefore, the exact mechanism behind the rescue effect of miR-4758 is yet to be elucidated and requires further investigation.

The upregulation of *CDKN1A* in GGs along with the slow growing phenotype of GGs could potentially be attributed to the opposing oncogenic properties of miR-519d and miR-4758. It must be noted that based on our experiments we cannot exclude both miRNAs targeting other genes related to the PI3K/AKT3/P21 pathway affecting GG proliferation.

Overall, our results indicate that miR-519d and miR-4758 are potential regulators of the cell cycle and therefore could play a role in formation of GGs. Furthermore, we showed that these miRNAs could be used as markers to distinguish GGs from DNTs and other low and high grade gliomas, which has implications for determining targeted therapeutic strategies and could prevent unnecessary treatment with chemo-radiation therapy. However, to determine the diagnostic value of these molecular markers in distinguishing GGs from DNTs, these miRNAs need to be validated in larger multicenter cohorts that include difficult-to-classify LEAT subtypes.

## MATERIALS AND METHODS

### Subjects

The majority of the cases included in this study were obtained from the archives of the Academic Medical Center of Amsterdam and the University Medical Center Utrecht. A further DNT case was included from the University College London Institute of Neurology. SEGA tissue was obtained from the following institutes: University Hospital Erlangen, Children's Memorial Health Institute in Warsaw, Hacettepe University in Ankara, and the University Hospital de Santa Maria (CHLN) in Lisbon. A total of 99 surgical tumour specimens were examined (Table 1; n=35 GNTs (WHO grade I); n= 64 astrocytomas; SEGA (*TSC1*/*TSC2* mutated) n=10; PA (WHO I) n=15; AII (*IDH-1* mutant) n=10; AIII (*IDH-1* mutant) n=14 and GB (*IDH-1* WT) n=15). Eight surgical specimens contained sufficient amount of peritumoural tissue (normal-appearing cortex/white matter adjacent to the tumour; Table 1). All tumour cases were reviewed independently by eight neuropathologists, and the diagnosis was confirmed according to the revised WHO classification of tumours of the central nervous system [1, 7]; only entities with consensus agreement between the neuropathologists were included (CD34 positive GGs and CD34 negative DNTs, all IDH1 negative). Additional molecular diagnosis was performed on GNT samples with sufficient amounts of DNA to identify mutations in *BRAF* and *FGFR1* genes. Control cortical specimens were obtained at autopsy from 17 patients. For the cortical specimens attention was taken to provide equal grey/white matter tissue components. For control tissue, all autopsies were performed within 24 hours after death. Tissue was obtained and used in accordance with the Declaration of Helsinki and the AMC Research Code provided by the Medical Ethics Committee and approved by the science committee of the UMC Utrecht Biobank.

**Table 1:** Summary of clinical findings of glioneuronal tumours (GNTs)

Patient	Diagnose	Gender	Localization	Age of surgery (years)	Age of seizure onset (years)	Duration of epilepsy (years)	Pre-operative seizure frequency (per month)	Post-operative outcome (Engel's score)	Mutation
1^a,c^	GG	F	T	29	13	16	200	1	*BRAF V600E*
2^a,c^	GG	F	T	11	8	3	56	1	*BRAF V600E*
3^a,c^	GG	M	T	16	14	2	30	1	*BRAF V600E*
4^a,c^	GG	F	T	42	11	31	60	1	NMI
5^a,b,c^	GG	M	T	21	13	8	70	2	*BRAF V600E*
6^a,c^	GG	F	T	14	11	3	55	1	*BRAF T559R* and BRAF V600E^*^
7^a,b^	GG	F	P	24	24	0	3	1	*BRAF V600E*
8^a,c^	GG	F	T	23	14	9	60	1	*BRAF V600E*
9^a^	GG	F	T	19	15	4	70	1	NMI
10^a^	GG	M	T	18	13	5	20	1	*BRAF V600E*
11^a,b^	GG	M	P	28	28	0	80	1	*BRAF V600E*
12^a^	GG	F	T	34	11	23	90	1	NMI
13^a^	GG	M	T	20	11	9	160	1	*BRAF V600E*
14^a^	GG	F	T	49	19	30	150	1	*BRAF V600E*
15^b^	GG	F	T	28	12	16	30	2	*BRAF V600E*
16^b^	GG	M	T	10	9	1	30	1	*BRAF V600E*
17^b^	GG	M	T	18	17	1	<5	1	NMI
18^b^	GG	M	T	44	20	24	10	1	NMI
19^b^	GG	F	T	6	3	3	10-20	1	NMI
20^b^	GG	M	P	42	1	41	>30	1	NMI
21^b^	GG	F	B	29	No seizures	--	--	--	NMI
22^b^	GG	M	T	33	18	15	--	1	NMI
23^b^	GG	F	T	22	10	12	--	1	NMI
24^b^	GG	F	C	48	No seizures	--	--	--	*BRAF V600E*
25^b^	GG	M	T	34	16	22	<5	1	*BRAF V600E*
26^b^	GG	F	T	56	52	4	80-100	2	*BRAF V600E*
27^a^	DNT	F	T	15	11	4	>50	1	NMI
28^a^	DNT	M	T	41	19	22	<5	1	NMI
29^a^	DNT	M	T	11	10	1	10-20	1	Mutation unknown
30^a^	DNT	M	T	8	2	6	>30	1	NMI
31^a^	DNT	F	T	30	25	5	5-10	4	*BRAF V600E*
32^a^	DNT	F	F	11	8	3	10-20	1	*CIC G935R*
33^a^	DNT	F	T	16	14	2	<5	1	NMI
34^a^	DNT	M	T	35	17	18	>30	1	*FGFR1 G539R* and *FGFR1 K656E*
35^a^	DNT	F	T	15	5	10	>50	1	NMI

M male, F female, F frontal, T temporal, P parietal, B brainstem, C cerebellum, NMI no mutation identified; ^a^FFPE (formalin-fixed, paraffin-embedded) tissue; ^b^Frozen tissue; ^c^Peritumoural cortex tissue. Gangliogliomas (GG) and dysembryoplastic neuroepithelial tumours (DNT). ^*^Both mutations are on the same allel.

### Mutation analysis

GG samples were screened using sanger sequencing as described previously [41]. Next generation sequencing (NGS) was performed on 10 GGs and 8 DNTs using a customised Ion AmpliSeq™ Neurology Panel (ThermoFisher Scientific, Waltham, Massachusetts, USA) for targeted multi-gene amplification, as previously reported [18]. This panel consists of the following genes; *AKT1*, *AKT3*, *ATRX*, *BRAF*, *CDK6*, *CIC*, *CTNNB1*, *DDX3X*, *DEPDC5*, *EZH2*, *FGFR1* (exon 12 and exon 14), *FUBP1*, *H3F3A*, *HIST1H3b*, *HIST1H3c*, *IDH1*, *IDH2*, *KDM6A*, *mTOR*, *MYB*, *MYBL1*, *NPRL2*, *NPRL3*, *PIK3CA*, *PIK3R1*, *PIK3R2*, *PTCH1*, *PTEN*, *SMARCA4*, *SMARCB1*, *SMO*, *SUFU*, *TP53*. *FGFR1* mutations below 5%, duplications and fusion genes cannot be detected with this panel.

### Tissue preparation

Brain tissue from control and tumour patients was fixed in 10% buffered formalin and embedded in paraffin. Paraffin-embedded tissue was sectioned at 5 μm and mounted on pre-coated glass slides (Star Frost, Waldemar Knittel GmbH, Brunschweig, Germany). Sections of all specimens were processed for hematoxylin and eosin (HE), staining as well as for immunohistochemical stainings for a number of neuronal and glial markers to confirm the diagnosis. Additional tissue from controls (n=8) and from patients with GG (n=15), PA (n=15), AII (n=10), AIII (n=14) and GB (n=15) was snap frozen in liquid nitrogen and stored at -80°C until further use (RNA isolation for microRNA array and/or quantitative real-time PCR).

### *In situ* hybridization

ISH for miR-519d and miR-4758 was performed using 5′ - 3′ double digoxygenin (DIG)-labeled Superior probes (miR-519d-3p; DIG-CacTcuAaaGggAggCacTuuG-DIG; miR-4758-3p: DIG-GagGguGguCagCagGugGggCa-DIG; Ribotask ApS, Odense, Denmark). The hybridizations were done on 5 μm sections of paraffin-embedded materials as described previously [30]. The probes were hybridized at 58°C for 1 h, and the hybridization was detected with alkaline phosphatase (AP)-labeled anti-DIG (Roche Applied Science, Basel, Switzerland). NBT (nitro-blue tetrazolium chloride)/BCIP (5-bromo-4-chloro-3′-indolyphosphate p-toluidine salt) was used as chromogenic substrate for AP. Negative control assays were performed without probes and without primary antibody (sections were blank). For double-staining, combining immunohistochemistry with ISH, the sections were first processed for ISH and then processed for immunohistochemistry with glial fibrillary acidic protein (GFAP; monoclonal mouse, Sigma, St. Louis, Mo, USA; 1:4000), and NeuN (neuronal nuclear protein; mouse clone MAB377; Chemicon, Temecula, CA, USA; 1:2000). Signal was detected using the chromogen 3-amino-9-ethylcarbazole (Sigma-Aldrich, St. Louis, MO, USA).

### RNA isolation

For RNA isolation, cells or frozen tissue were homogenized in Qiazol Lysis Reagent (Qiagen Benelux, Venlo, The Netherlands). Total RNA, including the miRNA fraction, was isolated using the miRNeasy Mini kit (Qiagen Benelux, Venlo, the Netherlands) according to manufacturer's instructions. The concentration and purity of RNA were determined using a Nanodrop spectrophotometer (Thermo Fisher Scientific, Wilmington, DE, USA). FFPE material was processed for RNA isolation using QuickExtract™ FFPE RNA Extraction Kit (Epicentre, Madison, WI, USA) according to manufacturer's instructions. The concentration of RNA was determined using a Qubit® 2.0 Fluorometer (Life Technologies, Carlsbad, CA, USA).

### MicroRNA microarrays

A screening for miRNAs was performed using the miRCURY LNA™ microRNA array (7th gen, Cat # 208500, Exiqon, Vedbaek, Denmark) by the Exiqon miRNA array service. Briefly, 5 μg of total RNA from 6 GG and 5 control cortex samples were labelled using the miRCURY LNA™ microRNA Hi-Power Labeling Kit, Hy3™/Hy5™ and hybridized on the miRCURY LNA™ microRNA Array. After washing, the slides were scanned and analysed using ImaGene^®^ 9 (miRCURY LNA™ microRNA Array Analysis Software, Exiqon, Vedbaek, Denmark). The quantified signals were corrected for background (Normexp with offset value 10 [42]), and normalized using the global Lowess (Locally Weighted Scatterplot Smoothing) regression algorithm.

### MicroRNA target prediction

Using the online database miRWalk (version 2.0; http://zmf.umm.uni-heidelberg.de/apps/zmf/mirwalk/) a total of 5 different databases (miRWalk, Microt4, miRanda, miRDB and Targetscan) were used to identify predicted targets of miR-519d and miR-4758. Targets were considered relevant if found in at least 3 of the 5 databases.

### Quantitative real-time PCR analysis

miRNA (miR-519d-3p, miR-4758-3p, miR-664b-3p, miR-4714-5p, miR-5681b and a reference gene, U6B small nuclear RNA gene, Rnu6B) expression was analyzed using Taqman microRNA assays (Applied Biosystems, Foster City, CA, USA). cDNA was generated using the Taqman MicroRNA reverse transcription kit (Applied Biosystems, Foster City, CA, USA) according to the manufacturer's instructions, and the PCRs were run on a Roche Lightcycler 480 thermocycler (Roche Applied Science, Basel, Switzerland).

To evaluate miRNA targets (*TIMP2*, *AKT3*, *ERBB3*, *ERBB4*, *PI3KCA*, *TP53*, *MDM2*, *CDK4*, *RB1*, *CDK2*, *CDKN1A* (P21), *CDKN1B* (P27), *JAK1 and PTEN*), 1 μg of cell culture derived total RNA or 500 ng of human brain material-derived total RNA were reverse-transcribed into cDNA using oligo dT primers (Supplementary Table 1). *EF1α* was used as a reference gene. PCRs were run as described previously [43] on a Roche Lightcycler 480 thermocycler (Roche Applied Science, Basel, Switzerland).

Quantification was performed using the computer program LinRegPCR in which linear regression on the Log (fluorescence) per cycle number data is applied to determine the amplification efficiency per sample [44, 45]. The starting concentration of each specific product was divided by the starting concentration of reference genes and this ratio was compared between groups.

### Cell transfection

The human pediatric low grade astrocytoma cell line (WHO grade II: Res-259; LGG2) was kindly provided by Dr Chris Jones (Institute of Cancer Research, Sutton, UK). Cells were cultured in Dulbecco's Modified Eagle's Medium (DMEM)/HAM F10 (1:1) (Gibco, Life Technology) supplemented with 50 units/ml penicillin, 50 μg/ml streptomycin and 10% fetal calf serum (FCS) in a humidifier incubator at 37°C with 5% CO_2_. Oligonucleotides were delivered to the cells using Lipofectamine® 2000 transfection reagent (Life Technologies, Grand Island, NY, USA) in a final concentration of 50 nM for a total of 24 hours. Cells were transfected with mimic pre-miRNA (Applied Biosystems, Carlsbad, CA, USA) for miR-519d-3p and/or miR-4758-3p. Cells treated with lipofectamine without mimic were used as a control. Cells were washed twice with PBS before harvesting.

### Cell-cycle analysis

At 24 hours after transfection, cells were collected and fixed in 90% ethanol at 4°C for another 24 hours. Fixed cells were then washed twice with PBS, resuspended in 200 μl PBS containing 1 mg/ml propidium iodide and 1 g/ml RNase and incubated for 10 min at 37°C. Cell cycle analysis was performed using the Fluorescent-Activated Cell Sorter Canto II (BD FACSCanto II, BD Biosciences, San Jose, CA, USA).

### Statistical analysis

Statistical analyses were performed with Graphpad Prism® software (Graphpad software Inc., La Jolla, CA, USA). Continuous variables were described with mean and ranges; categorical variables with proportions and percentages. The non-parametric Mann-Whitney test was used to assess differences between two groups. For multiple groups, the non-parametric Kruskal–Wallis test was used followed by a Mann-Whitney test to assess differences between groups. The spearman's rank correlation test was used to assess the correlation between miR-519d-3p and miR-4758-3p. Group differences and correlations were considered significant if p<0.05.

## SUPPLEMENTARY MATERIALS TABLE



## Abbreviations

AII: diffuse astrocytoma WHO grade II
AIII: diffuse astrocytoma WHO grade III
AP: alkaline phosphatase
AUC: area under the curve
DIG: double digoxygenin
DNTs: dysembryoplastic neuroepithelial tumours
FFPE: formalin-fixed paraffin-embedded
FCS: fetal calf serum
GB: glioblastoma
GGs: gangliogliomas
GNTs: glioneuronal tumours
HE: hematoxylin and eosin
ISH: *in situ* hybridization
LEATs: low-grade epilepsy-associated brain tumours
MAPK/ERK: mitogen-activated protein kinase/extracellular signal-regulated kinase
miRNAs: microRNAs
MVNT: multinodular and vacuolating neuronal tumours
NGS: next generation sequencing
PA: pilocytic astrocytomas
PI3K/AKT/mTOR: phosphoinositide 3-kinase/Protein Kinase B/mechanistic target of rapamycin
PLNTY: polymorphous low-grade neuroepithelial tumours
PXA: pleomorphic xanthoastrocytoma
ROC: receiver operating characteristic
