# Supplementary material for: MicroRNA519d and microRNA4758 can identify gangliogliomas from dysembryoplastic neuroepithelial tumours and astrocytomas
Source: Oncotarget. 2018 Jun 15;9(46):28103–15. doi: 10.18632/oncotarget.25563 (PMC6021349; doi:10.18632/oncotarget.25563)
Supplement: Supplementary file 1 [file oncotarget-09-28103-s001.pdf]

MicroRNA519d and microRNA4758 can identify gangliogliomas from dysembryoplastic neuroepithelial tumours and astrocytomas

SUPPLEMENTARY MATERIALS

Supplementary Table 1: Primer sequences for quantitative real-time PCR

Gene	Sequence (5'-3')
TIMP2	TGCAGATGTAGTGATCAGGGC
	TCTCAGGCCCTTTGAACATC
AKT3	ATTATTGCAAAGGATGAAGTGGC
	CGGTCTTTTGTCTGGAAGGA
ERBB3	GAGAGACCCTCACTGCTG
	GATGGTACATGACAAGGTGC
ERBB4	AGGAGTGAAATTGGACACAGC
	TCTCGGTATACAAACTGGTTTCC
p21 (CDKN1A)	CCTCCCCCTTGTCCTTTC
	GTGGGACAGGCACCTCAG
p27 (CDKN1B)	CCGGCTAACTCTGAGGACAC
	TTGCAGGTCGCTTCCTTATT
JAK1	GCATGAATAACATTTAAATTCCACA
	TGACTTTTTGGACAGAACACAAAC
PTEN	CTGCGCTCAGTTCTCTCCTC
	GCGGCTCAACTCTCAAACTT
PI3KCA	TTGTGACCTTCGGCTTTTTC
	CGGTTGCCTACTGGTTCAAT
TP53	TGGACGGTGGCTCTAGACTT
	CTCCCATGTGCTCAAGACTG
MDM2	CATTGTCCATGGCAAAACAG
	GGCAGGGCTTATTCCTTTTC
CDK4	GGTACCACCGACTGCACTG
	GATGACTGGCCTCGAGATGT
RB1	CAAAGCAGAAGGCAACTTGA
	GGGATTCCATGATTCGATGT
CDK2	TCCTCCACCGAGACCTTAAA
	CAGGGACTCCAAAAGCTCTG
EF1α	ATCCACCTTTGGGTCGCTTT
	CCGCAACTGTCTGTCTCATATCAC
